# Multi-omics mechanical analysis of gut microbiota, carboxylic acids, and cardiac gene expression interaction triggering diabetic cardiomyopathy

**DOI:** 10.1128/msystems.01450-24

**Published:** 2024-11-29

**Authors:** Meixin Shi, Bingbing Zhao, Wenjie Cai, Hui Yuan, Xiao Liang, Zhitao Li, Xinyu Liu, Ye Jin, Xi Liu, Can Wei

**Affiliations:** 1 Department of Pathophysiology, Harbin Medical University, Harbin, Heilongjiang, China; 2 Department of General Surgery, Key Laboratory of Hepatosplenic Surgery, Ministry of Education, The First Affiliated Hospital of Harbin Medical University, Harbin, Heilongjiang, China; 3 School of Basic Medical Sciences, Mudanjiang Medical University, Mudanjiang, Heilongjiang, China; 4 Department of Cardiology, The First Affiliated Hospital of Harbin Medical University, Harbin, Heilongjiang, China; 5 Department of Cardiology, Ordos Central Hospital, Ordos, China; 6 Department of Pathophysiology, Harbin Medical University, Harbin, Heilongjiang, China; University of California San Diego, La Jolla, California, USA

**Keywords:** diabetic cardiomyopathy, gut microbiota, PPAR signaling pathway, carboxylic acids

## Abstract

It is well known that gut microbial imbalance is a potential factor for the occurrence and development of diabetes mellitus (DM) and its complications. Moreover, the heart and gut microbiota can regulate each other through the gut–metabolite–heart axis. In this study, metagenomics, metabolomics, and transcriptomics were chosen to sequence the changes in gut microbiota, serum metabolite levels, and differentially expressed genes (DEGs) in leptin receptor-deficient db/db mice and analyze the correlation between serum metabolites and gut microbiota or DEGs. According to the results, there were significant differences in the 1,029 cardiac genes and 353 serum metabolites in diabetic mice of the db/db group, including DEGs enriched in the PPAR signaling pathway and increased short-chain carboxylic acids (CAs), when compared with the normal db/m group. According to metagenomics, the gut microbiota of mice in the db/db group were disrupted, and particularly Lachnospiraceae bacteria and Oscillospiraceae bacteria significantly decreased. Also, according to the Pearson correlation analysis, a significant positive correlation was found between CAs and PPAR signaling pathway-related DEGs, and a negative correlation was found between CAs and the abundance of the above-mentioned species. To sum up, type 2 diabetes mellitus (T2DM) can upregulate the expression of partial cardiac genes through the levels of serum short-chain CAs affected by gut microbiota, thus playing a role in the occurrence and development of diabetic cardiomyopathy (DCM).

**IMPORTANCE:**

Our research results clearly link the changes in heart genes of T2DM and normal mice with changes in serum metabolites and gut microbiota, indicating that some genes in biological processes are closely related to the reduction of protective microbiota in the gut microbiota. This study provides a theoretical basis for investigating the mechanism of DCM and may provide preliminary evidence for the future use of gut microbiota therapy for DCM.

## INTRODUCTION

As we all know, diabetes mellitus (DM) has imposed an enormous burden on the society, whether in terms of the expensive medical costs or the poor health status of patients (1). As estimated by *the International Diabetes Federation Diabetes Atlas*, its global prevalence rate has increased from 10.8% to 12.2%, and the number of adults with DM will increase from 536.6 million in 2021 to 783.2 million in 2045 (2), wherein over 90% of cases belong to type 2 diabetes mellitus (T2DM) (3). As a complication induced by DM, diabetic cardiomyopathy (DCM) initially presented as an isolated diastolic dysfunction, but will gradually develop into systolic dysfunction over time (4, 5). Cardiovascular complications, mainly ischemic heart disease, serve as the main cause of morbidity and mortality in patients with DM (6). Therefore, it is very significant to explore the mechanism of DCM.

With the progress of sequencing technology and bioinformatics, numerous reports have emerged regarding changes in the composition of disease-related gut microbiota, yet there are few reports on the causal relationship (7). Also, studies have found that changes in the abundance of gut microbiota were significantly associated with the development of DM (8). By altering the integrity of proinflammatory markers and the intestinal barrier, some gut microbiota can promote or retard the development of DM. Moreover, diabetes drugs such as metformin can also alter the composition of gut microbiota (9). The gut–heart axis represents a bilateral relationship between gut microbiota and the heart (10). As reported, myocardial ischemia–reperfusion injury can induce gut microbiota disorders and intestinal destruction, leading to bacterial translocation. Simultaneously, these changes can exacerbate myocardial injury through enhanced validation, in turn (11). In addition, the imbalance of gut microbiota is significantly correlated with an increase in systemic inflammation levels, which may be the pathogenesis of heart failure (12). However, the influence of the gut–heart axis on DCM still needs to be explored further.

So far, the gut microbiota has mainly exerted its role in cardiovascular diseases through two pathways (7). The first is intestinal leakage. Impaired intestinal barrier function may lead to the transfer of bacterial products to the host circulation, resulting in a proinflammatory state (7). The second is metabolites. The metabolic potential has been identified to contribute to the development of cardiovascular disease (13). By supplementing specific gut microbiota such as *Bifidobacterium longum*, *Lactobacillus acidophilus,* and *Enterococcus faecalis* to lower the plasma kynurenine levels, ventricular remodeling can be alleviated (14). Through lipopolysaccharide and glucose-induced activation of the NLRP3-inflammasome, gut microbiota dysbiosis can promote age-related atrial fibrillation (15).

In this study, we explored and analyzed the cardiac transcriptome, plasma metabolomics, and gut microbiota of genetically diabetic db/db (leptin receptor gene-deficient) mice. From the perspective of immunity, we investigated the gut metabolism–heart axis to demonstrate DCM. Our findings provide a theoretical basis and unique insights for studying the mechanism of the gut axis and metabolome in the occurrence and progression of DCM.

## RESULTS

### T2DM regulated the immune system process in mouse heart

**Fig 1:**
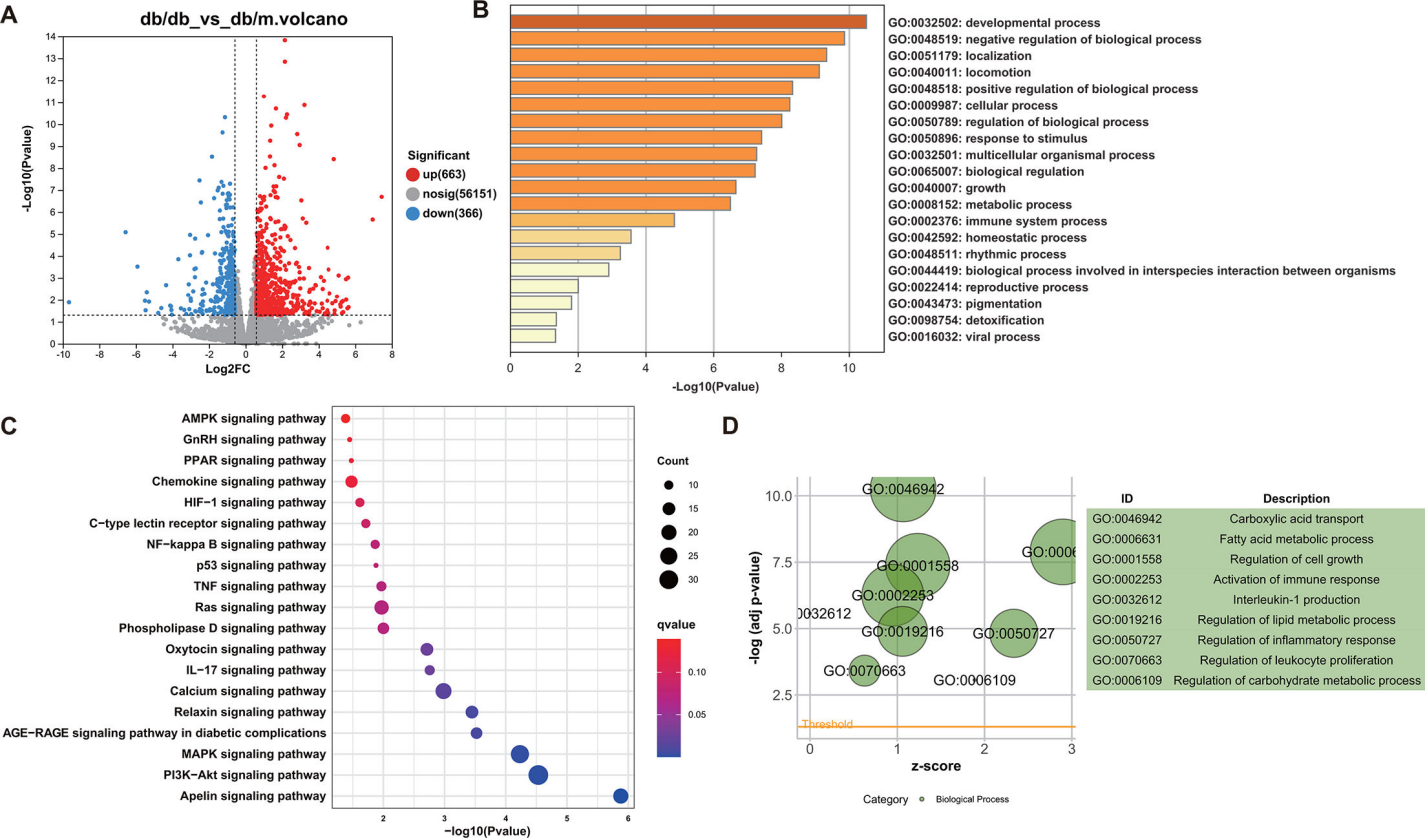
Functional analysis of the influences of T2DM on the mouse heart transcriptome. (**A**) Volcano plot of significant DEGs regulated between the db/m group and the db/db groups. (**B**) Column graphs of the top-level GO biological processes. (**C**) Bubble plots of KEGG pathway analysis. (**D**) Bubble plots of GO biological processes.

To investigate the occurrence and development mechanisms of DCM, RNA sequencing was performed on the heart of the db/m (heterozygous, leptin receptor gene-deficient) and db/db groups. The results of HE staining revealed that the morphological structure of the myocardial tissue in the db/db mice was significantly disorganized compared with that of the db/m group (Fig. S1). The volcano plot showed a total of 1,029 differentially expressed genes (DEGs) with a 1.5-fold difference between the db/m group and the db/db group, including 663 upregulated genes and 366 downregulated genes (Fig. 1A). The gene ontology (GO) analysis indicated that these genes were involved in the immune system process (Fig. 1B). To better understand how these DEGs promoted the DCM progress, the gene ontology_biological process (GO_BP) and Kyoto Encyclopedia of Genes and Genomes (KEGG) enrichment analysis were performed. The KEGG was mainly enriched as tumor necrosis factor (TNF), interleukin-17 (IL-17), nuclear factor kappa-B (NF-kappa B), and peroxisome proliferator-activated receptor (PPAR) signaling pathway (Fig. 1C; Table S1). The GO_BP was enriched in metabolic and immune-related biological processes, such as carboxylic acid transport, organic acid transport, fatty acid metabolic process, activation of immune response, and regulation of inflammatory response (Fig. 1D; Table S2). Taken together, these results indicated that T2DM might play a vital role in the immune system process as well as immune-related pathways.

### Serum metabolomes were changed in T2DM-induced mice

**Fig 2:**
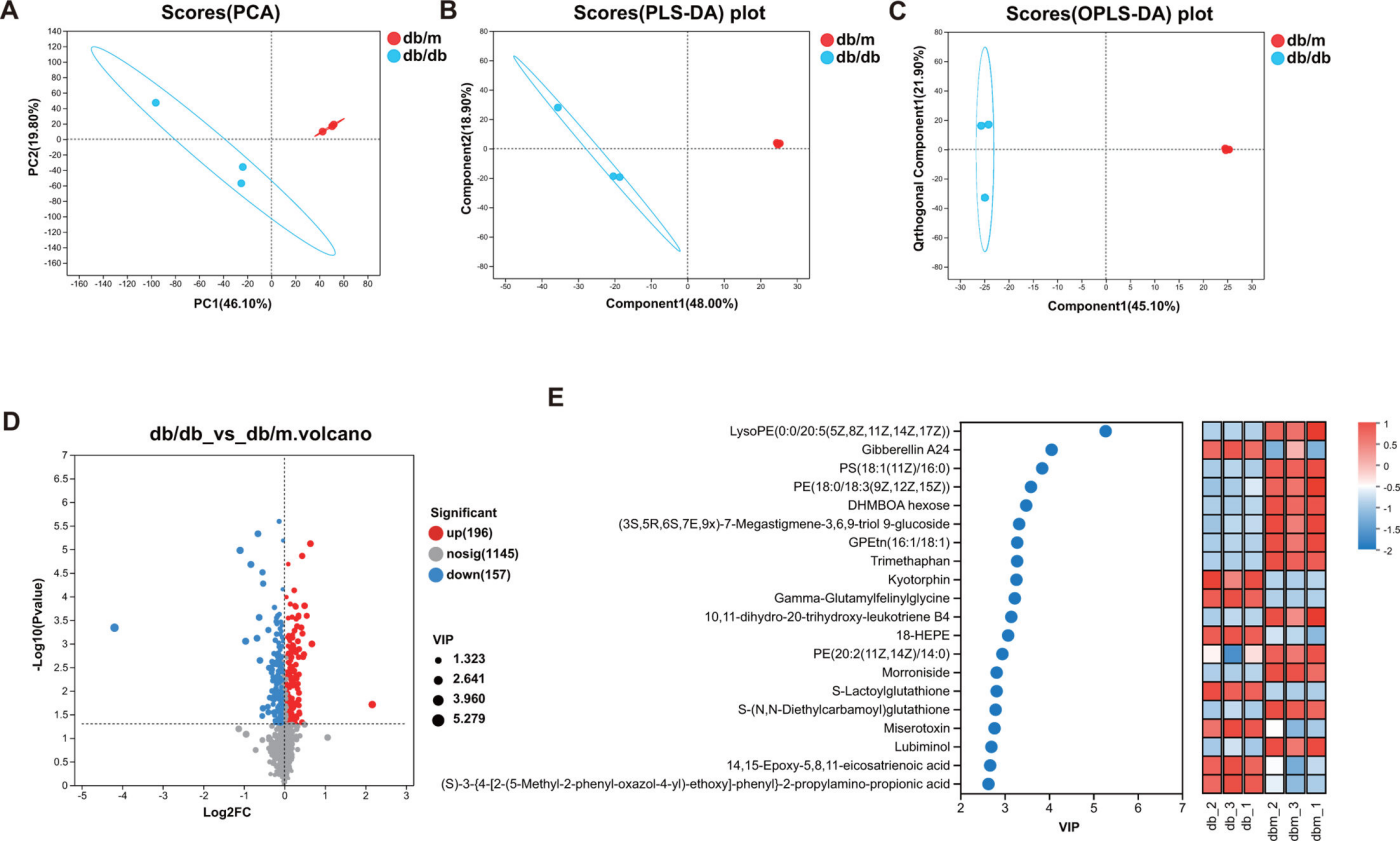
Effects of T2DM on the mouse heart metabolome. (**A**) PCA, (**B**) PLS-DA, and (**C**) OPLS-DA of myocardial metabolomics; (**D**) volcanic map of metabolomes between db/db vs db/m groups; (**E**) top 20 differential metabolites were identified between db/db vs db/m groups (VIP >1).

To evaluate the levels of metabolites in T2DM-induced cardiomyopathy, we performed metabolomics analysis on serum samples. First, we conducted a principal component analysis (PCA) on the entire study population, and partial least squares discriminant analysis (PLS-DA) and orthogonal partial least squares discriminant analysis (OPLS-DA) showed significant differences between the db/m group and the db/db group (Fig. 2A through C). A total of 353 differential metabolites, including 196 upregulated metabolites and 157 downregulated ones, were identified in the serum of the two groups through tandem mass spectrometry (MS/MS) analysis (Fig. 2D). The top 20 that contributed the most to the difference included gibberellin A24, trimethaphan, and 4-hydroxyretinoic acid (Fig. 2E; Table S3). The KEGG enrichment analysis revealed that these differential metabolites were mainly involved in carbohydrate digestion and absorption, glycerophospholipid metabolism, galactose metabolism, taste transduction, retrograde endocannabinoid signaling, insulin resistance, advanced glycation end-product (AGE) receptor for advanced glycation end-product (RAGE) signaling pathway in diabetic complications, cholesterol metabolism, diabetic cardiomyopathy, etc. (Fig. S2)

### Diabetic phenotype altered the diversity and composition of gut microbiota

**Fig 3:**
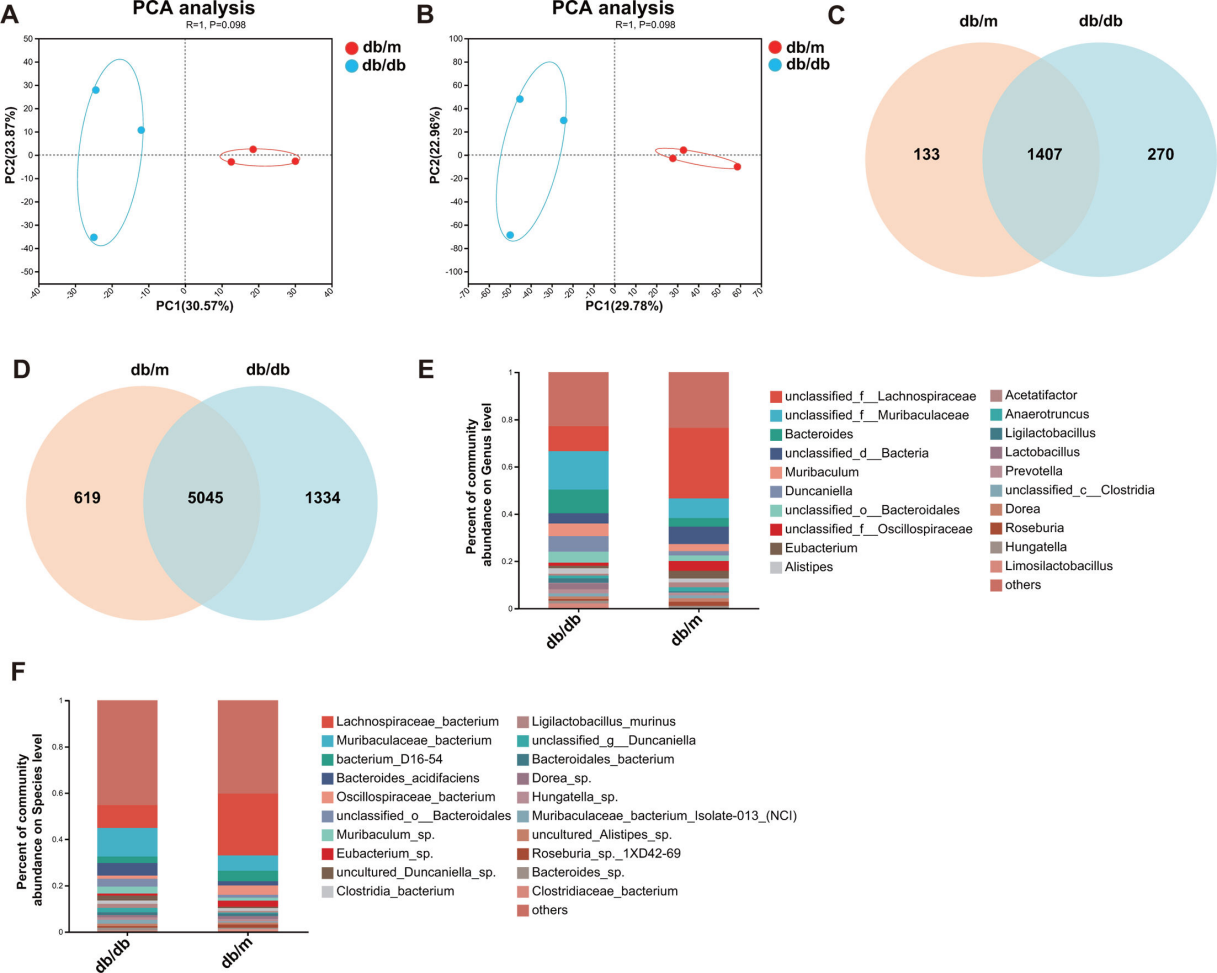
T2DM significantly altered the gut microbiota structure and composition. (**A**) Multiple-sample PCA of the genus; (**B**) multiple-sample PCA of the species; (**C and D**) stacked bar graphs of the relative abundance at the genus and species levels.

To investigate the influence of the diabetic phenotype on gut microbiota, metagenome sequencing was performed on gut content samples obtained from db/m mice and db/db mice. PCA was carried out for β-diversity determination between the two groups, and evident separation of the gut microbiota was observed on the two-dimensional PCA plots (Fig. 3A and B). The richness of species (breakaway estimates) indicated an increase in the db/db group compared to the db/m group by α diversity (Chao) (Fig. S3). According to the Venn plot, there are significant differences between the two groups at the genus and species levels (Fig. 3C and D). The histograms demonstrated the gut microbiota community structure and the differences in the relative abundance of major gut microbiota at the genus and species levels. At the genus level, the microbial community is mainly composed of *unclassified_f__Lachnospiraceae*, *unclassified_f__Muribaculaceae*, *Bacteroides*, *unclassified_d__Bacteria,* and *Muribaculum* (Fig. 3E). At the species level, *Lachnospiraceae_bacterium*, *Muribaculaceae_bacterium*, *bacterium_D16-54*, *Bacteroides_acidifaciens*, and *Oscillospiraceae_bacterium* were the most abundant species in all groups (Fig. 3F).

**Fig 4:**
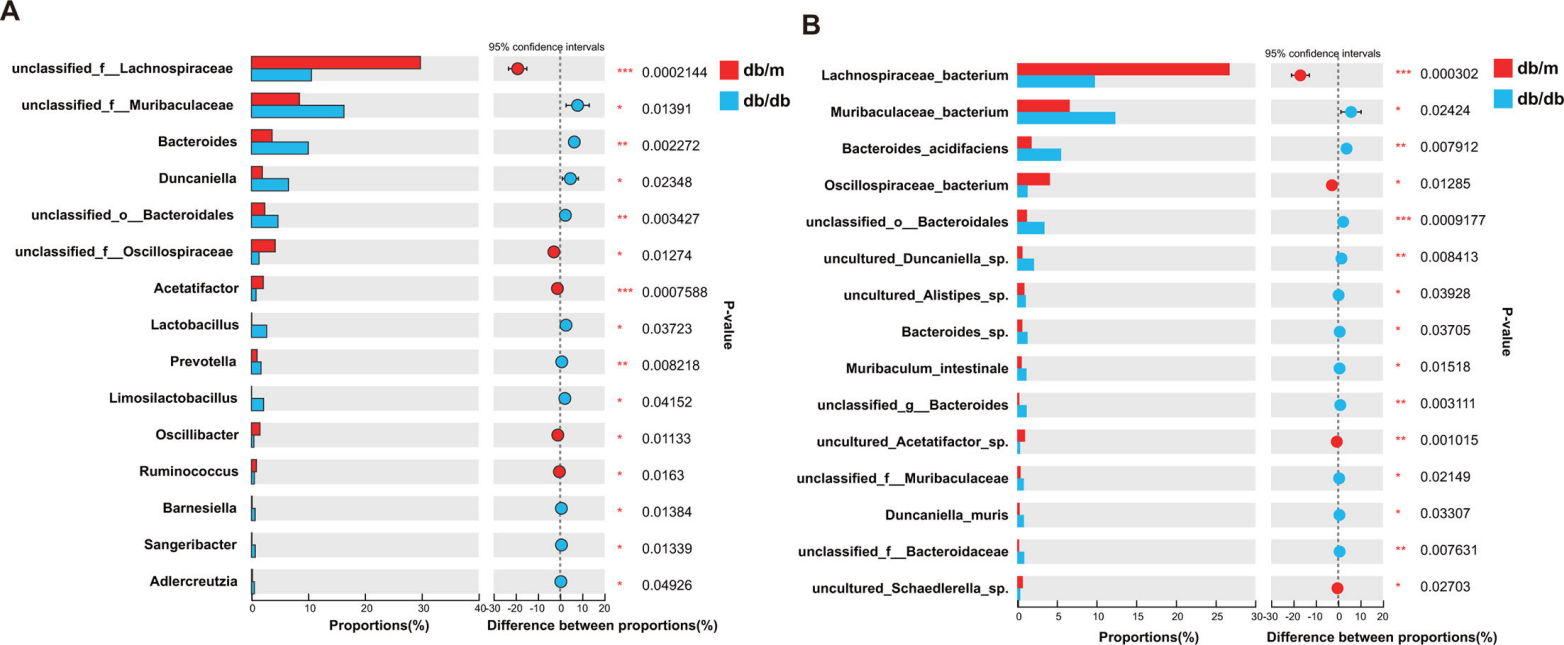
Differences in gut microorganisms based on genus and species abundance and functional enrichment among groups. (**A**) Column graphs of gut microbial differences at the genus level based on Student’s *t*-test; (**B**) Column graphs of gut microbial differences at the genus level based on Student’s *t*-test. * refers to the value of *P* < 0.05, and ** refers to the value of *P* < 0.001; the db/db group vs the db/m group.

Compared with those in the db/m group, mice in the db/db group showed significantly lower abundances of *unclassified_f__Lachnospiraceae* (6.93%) and higher abundances of *Bacteroides* (6.36%), *unclassified_f__Muribaculaceae* (7.89%), and *Duncaniella* (4.62%) at the genus levels (Fig. 4A). Correspondingly, at the species level, it was revealed that *Lachnospiraceae_bacterium* decreased by 16.95%, while *Muribaculaceae_bacterium* and *Bacteroides_acidifaciens* increased by 5.76% and 3.72%, respectively (Fig. 4B; Fig. S4). The above results indicated that there was a significant difference in microbial β diversity between mice in the db/m group and the db/db group. Moreover, pathogenic bacteria are significantly increased, while beneficial bacteria are significantly reduced in T2DM mice.

### Correlation analysis of DEGs, gut microbiota, and metabolites

**Fig 5:**
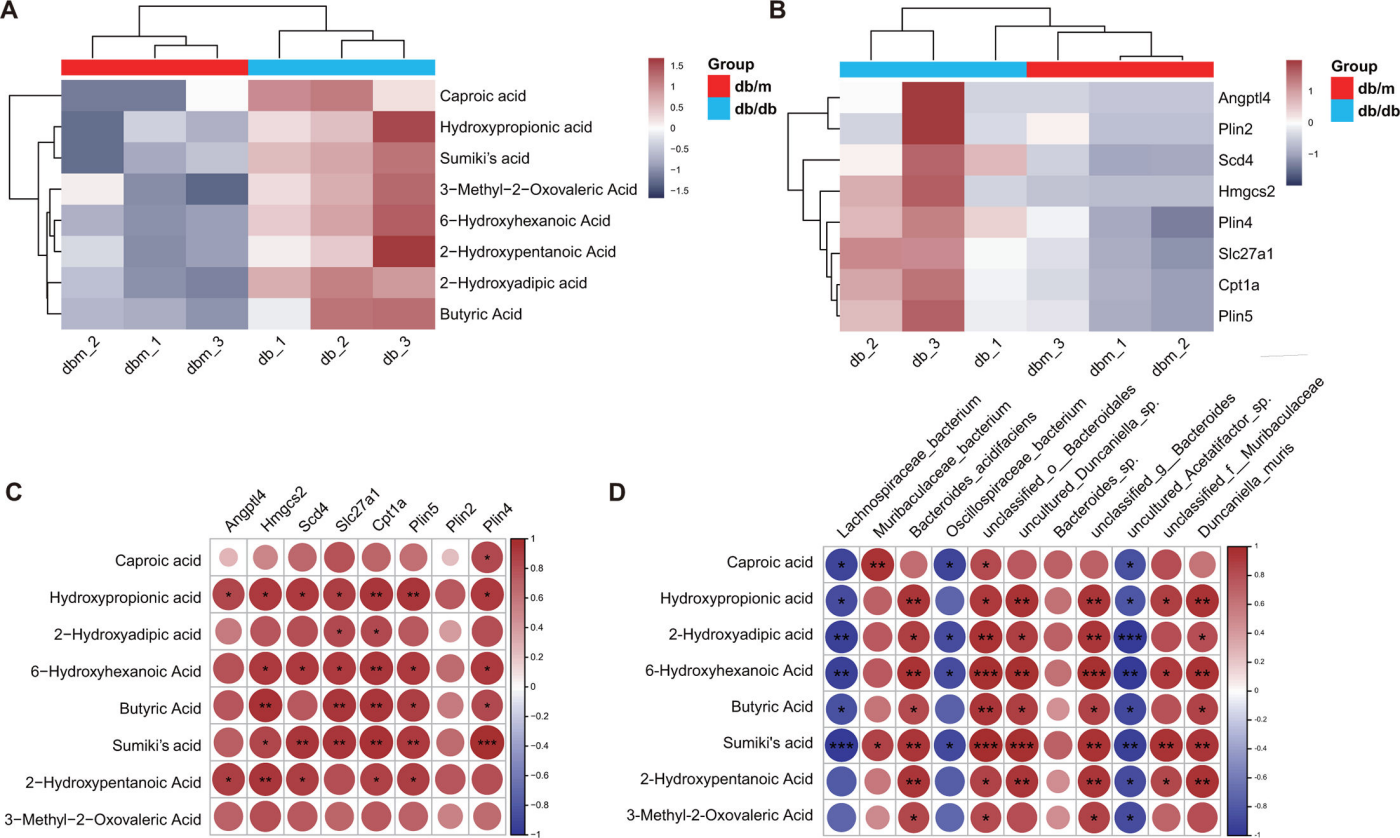
Statistical analysis using the Pearson correlation coefficient performed to examine the relationship between CAs, DEGs, and species. (**A**) Heatmap of CAs; (**B**) Heatmap of the DEGs in the PPAR signaling pathway; (**C**) heatmap of the Pearson correlation between CAs and PPAR signaling pathway-related DEGs; (**D**) heatmap of the Pearson correlation between CAs and microflora at the species level.

We focused on carboxylic acids with no more than six carbon atoms and significantly increased expression, including two short-chain fatty acids (SCFAs, namely, caproic acid and butyric acid) (Fig. 5A). The results demonstrated that all DEGs enriched in the PPAR signaling pathway are upregulated, including angiopoietin-like 4 (*Angptl4*), stearoyl-coenzyme A desaturase 4 (*Scd4*), 3-hydroxy-3-methylglutaryl-coenzyme A synthase 2 (*Hmgcs2*), solute carrier family 27 (fatty acid transporter), member 1 (*Slc27a1*), carnitine palmitoyltransferase 1 a (*Cpt1a*), as well as perilipin 2, 4, and 5 (*Plin2, 4,* and *5*) (Fig. 5B). The Sankey diagram reveals that, among the top 15 significantly different species, 11 species fall within the top 15 genera, including *Lachnospiraceae_bacterium*, *Muribaculaceae_bacterium*, *Bacteroides_acidifaciens,* and *Oscillospiraceae_bacterium* (Fig. S5). Pearson correlation analyses were performed on the above-mentioned DEGs, species, and CAs. The correlation between CAs and DEGs was analyzed in four inflammation-related pathways separately, and the results revealed that CAs were only significantly correlated with a small portion of DEGs enriched in the TNF, IL-17, and NF-kappa B signaling pathways. While all the CAs exhibited positive correlations with the DEGs enriched in the PPAR signaling pathway, including hydroxypropionic acid, 6-hydroxyhexanoic acid, butyric acid, Sumiki’s acid, and 2-hydroxypentanoic acid (Fig. S6; Fig. 5C). The relative abundance of *Lachnospiraceae_bacterium* and *Oscillospiraceae_bacterium* exhibited negative correlations with CAs. This finding contrasts with that of other species (Fig. 5D).

## DISCUSSION

As a global public health crisis, T2DM always poses a threat to the economies of all countries, especially developing ones. Over the years, with the change in living conditions, the diabetic population is getting younger and younger (16). As is known to all, cardiovascular disease serves as the main cause of death in patients with diabetes, including coronary artery disease and ischemic cardiomyopathy, which mainly presents impaired diastolic function (5). Persistent hyperglycemia induces hyperinsulinemia and insulin resistance, leading to abnormal glucose metabolism, myocardial cell apoptosis, and myocardial fibrosis, and the mechanisms involved include endoplasmic reticulum stress, oxidative stress, inflammation, calcium impaired processing, autophagy, mitochondrial autophagy, and renin–angiotensin–aldosterone system (17). Interestingly, recent studies have shown that the gut microbiota is closely associated with mechanisms that influence the development of DCM (18). Therefore, we constructed a genetically diabetic model using db/db mice and explored the role of the microbiome in T2DM from the perspectives of microbiome, metabolome, and transcriptome analyses.

T2DM is widely portrayed as a chronic, low-level metabolic inflammation and cellular stress state (19). Compared with normal mice without diabetes, we found 1,059 DEGs in the cardiac RNA of db/db mice, and some enriched in four inflammatory pathways, namely, the PPAR, NF-kappa B, TNF, and IL-17 signaling pathways. According to literature reports, in cardiovascular disease, PPARs can play an anti-inflammatory role by inhibiting proinflammatory pathways (20). These results indicate that the occurrence and development of DCM are regulated by the inflammatory pathway, PPAR signaling pathway.

Over the past decade, more and more shreds of evidence have demonstrated that the occurrence and development of T2DM are related to metabolite levels, such as those of amino acids, organic acids, bile acids, lipids, and acylcarnitine. (21) Upon comparing the abundances of serum metabolites in patients with diabetes and dilated cardiomyopathy, it was discovered that the organic heterocyclic compounds, oxygenated compounds, and nitrogen compounds involved in redox reactions, along with lipids and lipid molecules capable of regulating glucose and lipid metabolism, exhibited significant changes (22). The results of this study support the above conclusion. In this study, it was observed that the diversity and composition of the serum metabolites in mice with T2DM significantly increased, indicating eight increased short-chain organic acids (including two SCFAs). To explore the relationship between serum metabolites and host phenotype, Pearson’s correlation was employed to test the association between metabolites and DEGs, which showed that the above-mentioned carboxylic acids were significantly positively correlated with DEGs enriched in the PPAR signaling pathway, especially hydroxypropanoic acid and 6-hydroxyhexanoic acid, butyric Acid, and Sumiki’ s acid. This indicates that the occurrence of DCM is related to the mouse serum metabolism immune axis, especially the PPAR signaling pathway.

It has been reported that gut microbiota-related metabolites act as crucial intermediates in the crosstalk between the gut microbiota and host, significantly influencing the development of cardiometabolic diseases (23). So far, as the most extensively studied metabolite of gut microbiota, short-chain fatty acids control immune-regulatory function, promote intestinal epithelial integrity, and regulate insulin secretion and pancreatic beta cell proliferation, playing multiple roles in insulin resistance and T2DM (24). Therefore, it is of great significance to examine the influence of gut microbiota on health. In this study, it was observed that the diversity and composition of gut microbiota in mice with T2DM significantly increased. Moreover, a significant correlation was found between serum CA levels and several bacterial families associated with DCM, particularly those from Bacillota (formerly known as Firmicutes). Notably, there was a significant reduction *of Lachnospiraceae_bacterium, Oscillospiraceae_bacterium,* and *uncultured_Acetatifactor_sp* in DCM mice.

Firmicutes (Bacillota) is one of the two major phyla in the gut microbiota of healthy adults (25). *Lachnospiraceae_bacterium* is a protective symbiotic strain that produces short-chain fatty acids such as butyrate by fermenting dietary fiber (26). It has been reported that the abundance of *Lachnospiraceae_bacterium* was significantly decreased in patients with normal glucose tolerance, impaired glucose tolerance, and DM, indicating a negative correlation with DM (27). This result is in line with the findings of this study. Pearson’s correlation was employed to test the association between species and metabolic parameters, and the results indicated that *Lachnospiraceae_bacterium* is significantly negatively correlated with short-chain carboxylic acid (capronic acid, 2-hydroxyadipic acid, 6-hydroxyhexanoic acid, butyric acid, and Sumiki’s acid). This result indicates that *Lachnospiraceae_bacterium* can affect the expression of PPAR signaling pathway-related DEGs (including *Hmgcs2*, *Scd4*, *Slc27a1*, *Cpt1a*, *Plin5,* and *Plin4*) through organic acids (especially 6-hydroxyhexanoic acid, butyric acid, and Sumiki’s acid), thereby contributing to a hypoglycemic effect and inhibiting myocardial injury in T2DM. *Oscillospiraceae_bacterium* was found to be negatively correlated with CAs (capronic acid, 2-hydroxyadipic acid, 6-hydroxyhexanoic acid, and Sumiki’s acid). All of these indicate that the gut microbiota (especially Firmicutes) can regulate T2DM-induced myocardial injury by partially mediating the levels of short-chain CA metabolism.

**Fig 6:**
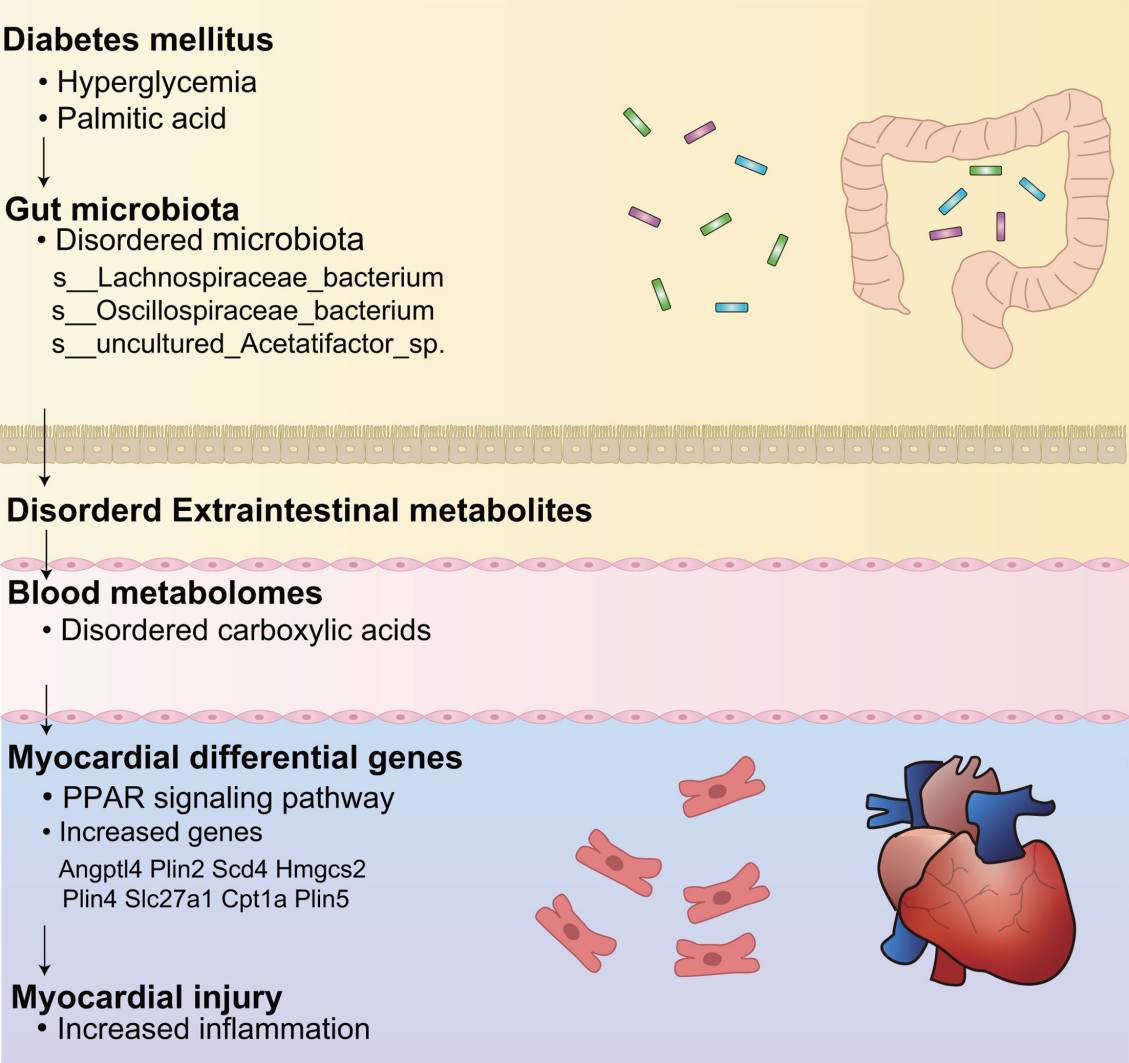
Schematic diagram of T2DM affecting myocardial injury through gut microbiota.

To sum up, this study has provided conceptual evidence for the main idea. These findings reflect the crosstalk among the cardiac gene expression, serum metabolite levels, and gut microbiota. It has been verified that the occurrence mechanism of DCM is partially mediated by reducing the levels of CAs regulated by protective bacteria (Firmicutes), further regulating the PPAR signaling pathway (Fig. 6). This explains the relationship between gut microbiota and the occurrence and development of DCM and provides theoretical basis and conceptual evidence for targeted gut microbiota therapy for DCM.

## MATERIALS AND METHODS

### Animals

The db/m mice (18–20 g, 6–8 weeks, male, specific pathogen-free grade, as the control group) and db/db mice (25–30 g, 6–8 weeks, male, specific pathogen-free grade) were provided by the Animal Center of Harbin Medical University (Harbin, China). All mice were treated following *the Guide for the Care and Use of Laboratory Animals* as adopted and promulgated by the US NIH. All treatment protocols were approved by the Institutional Animal Care and Use Committee at the Experimental Animal Center of Harbin Medical University (Harbin, Heilongjiang Province, China). The mice were raised on a normal diet under standard humidity and temperature for 24 weeks.

### Induction of gut contents, serum, and heart

After 16 weeks of modeling, the mice were anesthetized by intraperitoneal injection of 0.3% pentobarbital sodium (1 mg/20 g) and dissected under sterile conditions. The mouse heart was carefully dissected from surrounding tissues and cut into small pieces. A piece was randomly collected into sterile frozen test tubes, with each containing three mouse hearts. The sample was immediately frozen in liquid nitrogen; blood was drawn from the eye socket, placed into a sterile 1.5-mL EP tube, centrifuged at 3,000 rpm at 4°C for 15 minutes, and then serum samples were collected. Each tube contains serum from three mice; the intestine was separated from the body cavity using sterile scissors and forceps, and the contents of the large intestine were collected into sterile cryotubes. In addition, each test tube contains the gut contents of three mice and was immediately frozen in liquid nitrogen. All samples were stored at −80°C until further analysis.

### Transcriptome analysis

We collected heart samples from the db/m group and the db/db group at 24 weeks of modeling for sequencing (*n* = 3 per group, each sample contains heart tissues from three mice). Total RNA was extracted from tissue samples, the concentration and purity of RNA were checked by NanoDrop 2000, RNA integrity was checked by agarose gel electrophoresis, and RNA integrity number (RIN) values were determined using Agilent 2100. mRNA was enriched using Oligo DT magnetic beads and sequenced on the Illumina HiSeq 6000 platform. Differential expression analysis was performed using the DESeq2 R package. To explore the potential functions of DEGs, the Metascape and clusterProfiler R package were used to test the statistical enrichment of DEGs in GO and the KEGG. The R language was employed to generate volcano plots, circle plots, and bubble plots.

### Metagenomics statistics and bioinformatics analysis

The LC/MS analysis was performed on all serum samples. The R package (ropls) was used for PCA, PLS-DA, OPLS-DA, and differential metabolite analysis of serum samples. The KEGG functional pathway analysis was based on the KEGG keg_v20230830 analysis, and the HMDB compound classification was based on the HMDB (https://hmdb.ca). The R language was used to generate volcano plots, the KEGG bar charts, and the heat maps.

### Gut microbiota statistics and bioinformatics analysis

The DNA of gut contents was extracted and fragmented into approximately 350 base pairs. A library was constructed and amplified. Subsequently, the Illumina HiSeq platform (Illumina Inc., CA, USA) was further used for metagenomic sequencing. Fastp (https://github.com/OpenGene/fastp) was employed for quality control. Based on NR (ftp://ftp.ncbi.nlm.nih.gov/blast/db/), the sequence function was annotated. Diamond (https://github.com/bbuchfink/diamond) was utilized for comparing and analyzing annotations in multiple types of databases. The R stats package was employed to analyze species differences. R language was used to generate bar plots and Venn plots, and Circos-0.67–7 (http://circos.ca/) was used to generate Circos diagrams of sample and species, function, or gene relationship.

### Correlation analysis

First, Student’s *t*-test was chosen to confirm whether there were any differences in the relative abundance of microbial communities in the sample (*P < 0.05*). Then, the DEGseq2 R package was used to carry out transcriptome difference analysis (the threshold value is *P < 0.05* and |log2FC| > 0.59). Finally, Pearson’s association was established between serum metabolomics and cardiac transcriptome or serum metabolomics and gut microbiota metagenomics. |R| > 0.8 and *P* < 0.05, indicating a strong correlation.

## Data Availability

The raw reads of transcriptomics sequencing were deposited into the NCBI Sequence Read Archive (SRA) database (BioProject Number: PRJNA1119726).

## SUPPLEMENTAL MATERIAL

Supplemental Material msystems.01450-24-s0001.pdf Tables S1 to S3，Figures S1 to S6.

ASM does not own the copyrights to Supplemental Material that may be linked to, or accessed through, an article. The authors have granted ASM a non-exclusive, world-wide license to publish the Supplemental Material files. Please contact the corresponding author directly for reuse.

## References

[1] Zimmet P, Alberti KG, Magliano DJ, Bennett PH. 2016. Diabetes mellitus statistics on prevalence and mortality: facts and fallacies. Nat Rev Endocrinol 12:616–622. doi:10.1038/nrendo.2016.105 27388988

[2] Sun H, Saeedi P, Karuranga S, Pinkepank M, Ogurtsova K, Duncan BB, Stein C, Basit A, Chan JCN, Mbanya JC, Pavkov ME, Ramachandaran A, Wild SH, James S, Herman WH, Zhang P, Bommer C, Kuo S, Boyko EJ, Magliano DJ. 2022. IDF Diabetes Atlas: global, regional and country-level diabetes prevalence estimates for 2021 and projections for 2045. Diabetes Res Clin Pract 183:109119. doi:10.1016/j.diabres.2021.109119 34879977 PMC11057359

[3] Majety P, Lozada Orquera FA, Edem D, Hamdy O. 2023. Pharmacological approaches to the prevention of type 2 diabetes mellitus. Front Endocrinol (Lausanne) 14:1118848. doi:10.3389/fendo.2023.1118848 36967777 PMC10033948

[4] Tan Y, Zhang Z, Zheng C, Wintergerst KA, Keller BB, Cai L. 2020. Mechanisms of diabetic cardiomyopathy and potential therapeutic strategies: preclinical and clinical evidence. Nat Rev Cardiol 17:585–607. doi:10.1038/s41569-020-0339-2 32080423 PMC7849055

[5] Dillmann WH. 2019. Diabetic cardiomyopathy. Circ Res 124:1160–1162. doi:10.1161/CIRCRESAHA.118.314665 30973809 PMC6578576

[6] Bugger H, Abel ED. 2014. Molecular mechanisms of diabetic cardiomyopathy. Diabetologia 57:660–671. doi:10.1007/s00125-014-3171-6 24477973 PMC3969857

[7] Witkowski M, Weeks TL, Hazen SL. 2020. Gut microbiota and cardiovascular disease. Circ Res 127:553–570. doi:10.1161/CIRCRESAHA.120.316242 32762536 PMC7416843

[8] Gurung M, Li Z, You H, Rodrigues R, Jump DB, Morgun A, Shulzhenko N. 2020. Role of gut microbiota in type 2 diabetes pathophysiology. EBioMedicine 51:102590. doi:10.1016/j.ebiom.2019.11.051 31901868 PMC6948163

[9] Iatcu CO, Steen A, Covasa M. 2021. Gut microbiota and complications of type-2 diabetes. Nutrients 14:166. doi:10.3390/nu14010166 35011044 PMC8747253

[10] Trøseid M, Andersen GØ, Broch K, Hov JR. 2020. The gut microbiome in coronary artery disease and heart failure: current knowledge and future directions. EBioMedicine 52:102649. doi:10.1016/j.ebiom.2020.102649 32062353 PMC7016372

[11] Zhao J, Zhang Q, Cheng W, Dai Q, Wei Z, Guo M, Chen F, Qiao S, Hu J, Wang J, Chen H, Bao X, Mu D, Sun X, Xu B, Xie J. 2023. Heart-gut microbiota communication determines the severity of cardiac injury after myocardial ischaemia/reperfusion. Cardiovasc Res 119:1390–1402. doi:10.1093/cvr/cvad023 36715640 PMC10262181

[12] Madan S, Mehra MR. 2020. The heart-gut microbiome axis in advanced heart failure. J Heart Lung Transplant 39:891–893. doi:10.1016/j.healun.2020.04.003 32327221

[13] Tang WHW, Kitai T, Hazen SL. 2017. Gut microbiota in cardiovascular health and disease. Circ Res 120:1183–1196. doi:10.1161/CIRCRESAHA.117.309715 28360349 PMC5390330

[14] Shi B, Zhang X, Song Z, Dai Z, Luo K, Chen B, Zhou Z, Cui Y, Feng B, Zhu Z, Zheng J, Zhang H, He X. 2023. Targeting gut microbiota-derived kynurenine to predict and protect the remodeling of the pressure-overloaded young heart. Sci Adv 9:eadg7417. doi:10.1126/sciadv.adg7417 37450589 PMC10348671

[15] Zhang Y, Zhang S, Li B, Luo Y, Gong Y, Jin X, Zhang J, Zhou Y, Zhuo X, Wang Z, Zhao X, Han X, Gao Y, Yu H, Liang D, Zhao S, Sun D, Wang D, Xu W, Qu G, Bo W, Li D, Wu Y, Li Y. 2022. Gut microbiota dysbiosis promotes age-related atrial fibrillation by lipopolysaccharide and glucose-induced activation of NLRP3-inflammasome. Cardiovasc Res 118:785–797. doi:10.1093/cvr/cvab114 33757127

[16] Hu FB. 2011. Globalization of diabetes: the role of diet, lifestyle, and genes. Diabetes Care 34:1249–1257. doi:10.2337/dc11-0442 21617109 PMC3114340

[17] Huo J-L, Feng Q, Pan S, Fu W-J, Liu Z, Liu Z. 2023. Diabetic cardiomyopathy: early diagnostic biomarkers, pathogenetic mechanisms, and therapeutic interventions. Cell Death Discov 9:256. doi:10.1038/s41420-023-01553-4 37479697 PMC10362058

[18] Yuan S, Cai Z, Luan X, Wang H, Zhong Y, Deng L, Feng J. 2022. Gut microbiota: a new therapeutic target for diabetic cardiomyopathy. Front Pharmacol 13:963672. doi:10.3389/fphar.2022.963672 36091756 PMC9461091

[19] Tong A, Li Z, Liu X, Ge X, Zhao R, Liu B, Zhao L, Zhao C. 2024. Laminaria japonica polysaccharide alleviates type 2 diabetes by regulating the microbiota-gut-liver axis: a multi-omics mechanistic analysis. Int J Biol Macromol 258:128853. doi:10.1016/j.ijbiomac.2023.128853 38134985

[20] Montaigne D, Butruille L, Staels B. 2021. PPAR control of metabolism and cardiovascular functions. Nat Rev Cardiol 18:809–823. doi:10.1038/s41569-021-00569-6 34127848

[21] Chen ZZ, Gerszten RE. 2020. Metabolomics and proteomics in Type 2 diabetes. Circ Res 126:1613–1627. doi:10.1161/CIRCRESAHA.120.315898 32437301 PMC11118076

[22] Hao M, Deng J, Huang X, Li H, Ou H, Cai X, She J, Liu X, Chen L, Chen S, Liu W, Yan D. 2022. Metabonomic characteristics of myocardial diastolic dysfunction in type 2 diabetic cardiomyopathy patients. Front Physiol 13:863347. doi:10.3389/fphys.2022.863347 35651872 PMC9150260

[23] Deng K, Xu J-J, Shen L, Zhao H, Gou W, Xu F, Fu Y, Jiang Z, Shuai M, Li B-Y, Hu W, Zheng J-S, Chen Y-M. 2023. Comparison of fecal and blood metabolome reveals inconsistent associations of the gut microbiota with cardiometabolic diseases. Nat Commun 14:571. doi:10.1038/s41467-023-36256-y 36732517 PMC9894915

[24] Morrison DJ, Preston T. 2016. Formation of short chain fatty acids by the gut microbiota and their impact on human metabolism. Gut Microbes 7:189–200. doi:10.1080/19490976.2015.1134082 26963409 PMC4939913

[25] Sun Y, Zhang S, Nie Q, He H, Tan H, Geng F, Ji H, Hu J, Nie S. 2023. Gut firmicutes: relationship with dietary fiber and role in host homeostasis. Crit Rev Food Sci Nutr 63:12073–12088. doi:10.1080/10408398.2022.2098249 35822206

[26] Zhang X, Zhang Y, He Y, Zhu X, Ai Q, Shi Y. 2023. β-glucan protects against necrotizing enterocolitis in mice by inhibiting intestinal inflammation, improving the gut barrier, and modulating gut microbiota. J Transl Med 21:14. doi:10.1186/s12967-022-03866-x 36627673 PMC9830848

[27] Zhang B, Zhang X, Luo Z, Ren J, Yu X, Zhao H, Wang Y, Zhang W, Tian W, Wei X, Ding Q, Yang H, Jin Z, Tong X, Wang J, Zhao L. 2024. Microbiome and metabolome dysbiosis analysis in impaired glucose tolerance for the prediction of progression to diabetes mellitus. J Genet Genomics 51:75–86. doi:10.1016/j.jgg.2023.08.005 37652264

