# Erratum for Shi et al., “Multi-omics mechanical analysis of gut microbiota, carboxylic acids, and cardiac gene expression interaction triggering diabetic cardiomyopathy”

**DOI:** 10.1128/msystems.01138-25

**Published:** 2025-09-11

**Authors:** Meixin Shi, Bingbing Zhao, Wenjie Cai, Hui Yuan, Xiao Liang, Zhitao Li, Xinyu Liu, Ye Jin, Xi Liu, Can Wei

## ERRATUM

Volume 10, no. 1, e01450-24, 2025, https://doi.org/10.1128/msystems.01450-24. Page 5, Fig. 3 legend: The following should appear after “(C and D)”: Venn plot of the community differences at the genus and species levels; (E and F).

