# Supplementary material for: Multi-omics mechanical analysis of gut microbiota, carboxylic acids, and cardiac gene expression interaction triggering diabetic cardiomyopathy
Source: mSystems. 2024 Nov 29;10(1):e01450-24. doi: 10.1128/msystems.01450-24 (PMC11748484; doi:10.1128/msystems.01450-24)
Supplement: Supplemental Material — Tables S1 to S3，Figures S1 to S6. [file msystems.01450-24-s0001.pdf]

Supplementary materials

Table S1 KEGG pathways with statistical enrichment of DEGs

ID	Pathway	pvalue	qvalue	Count
mmu04371	Apelin signaling pathway	0.00000132	0.000310502	20
mmu04151	PI3K-Akt signaling pathway	0.00002938	0.001724119	33
mmu04010	MAPK signaling pathway	0.00005861	0.002751653	28
mmu04933	AGE-RAGE signaling pathway in diabetic complications	0.00029939	0.009286019	13
mmu04926	Relaxin signaling pathway	0.00035603	0.009286019	15
mmu04020	Calcium signaling pathway	0.00104045	0.01878699	22
mmu04657	IL-17 signaling pathway	0.00174116	0.02736709	11
mmu04921	Oxytocin signaling pathway	0.00193933	0.02736709	15
mmu04072	Phospholipase D signaling pathway	0.00998154	0.072107438	13
mmu04014	Ras signaling pathway	0.01067346	0.072107438	18
mmu04668	TNF signaling pathway	0.01075145	0.072107438	11
mmu04115	p53 signaling pathway	0.01315134	0.077177611	8
mmu04064	NF-kappa B signaling pathway	0.01356413	0.077394624	10
mmu04625	C-type lectin receptor signaling pathway	0.01933117	0.088975267	10
mmu04066	HIF-1 signaling pathway	0.02406397	0.102703663	10
mmu04062	Chemokine signaling pathway	0.03306757	0.124040788	14
mmu03320	PPAR signaling pathway	0.0332899	0.124040788	8
mmu04912	GnRH signaling pathway	0.03525502	0.127598764	8
mmu04152	AMPK signaling pathway	0.04129281	0.139092075	10

Table S2 GO_BP process with statistical enrichment of DEGs

ID	Description	pvalue	qvalue	Count
GO:0046942	Carboxylic acid transport	2.16439E-14	3.57866E-11	43
GO:0015849	Organic acid transport	2.62188E-14	3.57866E-11	43
GO:0006631	Fatty acid metabolic process	3.84808E-11	8.44879E-09	43
GO:0001558	Regulation of cell growth	2.0309E-10	2.76395E-08	42
GO:0002253	Activation of immune response	6.67618E-09	3.61109E-07	40
GO:0032612	Interleukin-1 production	4.88639E-08	1.74707E-06	18
GO:0019216	Regulation of lipid metabolic process	3.28601E-07	8.30151E-06	32
GO:0050727	Regulation of inflammatory response	4.04473E-07	9.86387E-06	31
GO:0070663	Regulation of leukocyte proliferation	1.86324E-05	0.000250894	23
GO:0006109	Regulation of carbohydrate metabolic process	0.000051282	0.00056516	18

Table S3 VIP value and fold change of top 20 differential metabolites

Metabolite	VIP_Oplsda	FC(dbdb/dbm)	pvalue	qvalue
LysoPE(0:0/20:5(5Z,8Z,11Z,14Z,17Z))	5.2793	0.0548	0.0004583	0.02563
Gibberellin A24	4.0607	4.4854	0.01948	0.1296
PS(18:1(11Z)/16:0)	3.8484	0.4685	0.00001053	0.00726
PE(18:0/18:3(9Z,12Z,15Z))	3.5954	0.5149	0.0008862	0.03286
DHMBOA hexose	3.4868	0.5634	0.00002093	0.009922
(3S,5R,6S,7E,9x)-7-Megastigmene-3,6,9-triol 9-glucoside	3.3282	0.6257	0.0007671	0.03031
GPEtn(16:1/18:1)	3.2849	0.6491	0.0002773	0.0251
Trimethaphan	3.2829	0.6357	0.00000468	0.0071
Kyotorphin	3.2675	1.5988	0.001011	0.03519
Gamma-Glutamylfelinylglycine	3.2302	1.5586	0.000007594	0.0072
10,11-dihydro-20-trihydroxy-leukotriene B4	3.1513	0.6577	0.002263	0.04765
18-HEPE	3.0799	1.4109	0.0001573	0.02131
PE(20:2(11Z,14Z)/14:0)	2.951	0.6929	0.02342	0.1381
Morroniside	2.8214	0.6947	0.00005335	0.01697
S-Lactoylglutathione	2.8212	1.3537	0.00001382	0.008066
S-(N,N-Diethylcarbamoyl)glutathione	2.7977	0.6872	0.00003075	0.01228
Miserotoxin	2.7742	1.395	0.001683	0.04333
Lubiminol	2.7029	0.7596	0.0005131	0.02648
14,15-Epoxy-5,8,11-eicosatrienoic acid	2.6751	1.376	0.001909	0.04503
(S)-3-{4-[2-(5-Methyl-2-phenyl-oxazol-4-yl)-ethoxy]-phenyl}-2-propylamino-propionic acid	2.637	1.3333	0.0004539	0.02563

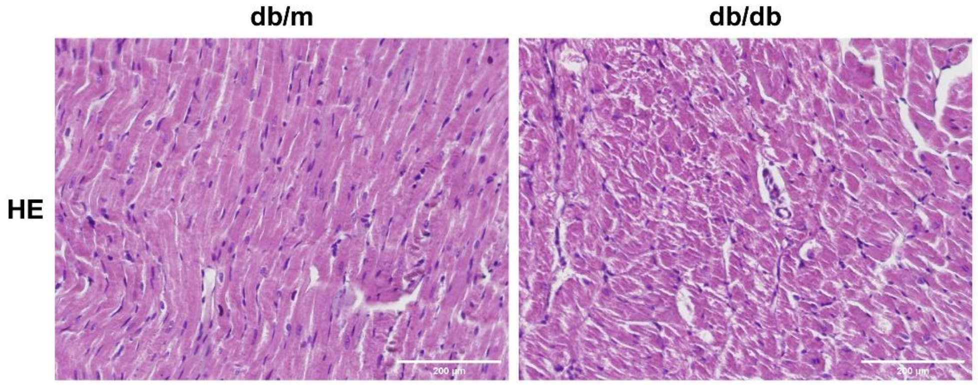

Fig. S1. Representative images of heart tissue stained with HE (Scar bar = 200μm)

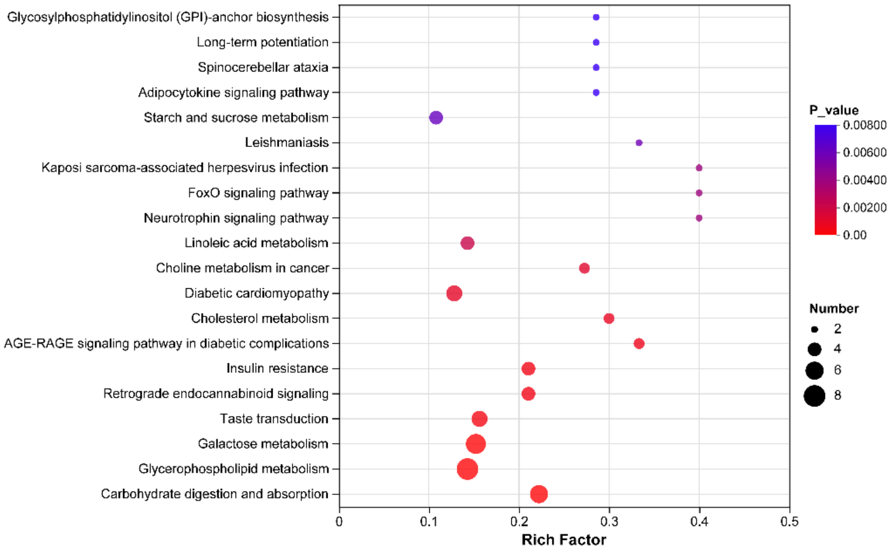

Fig. S2. Bubble plot describes KEGG enrichment analysis of differential metabolites

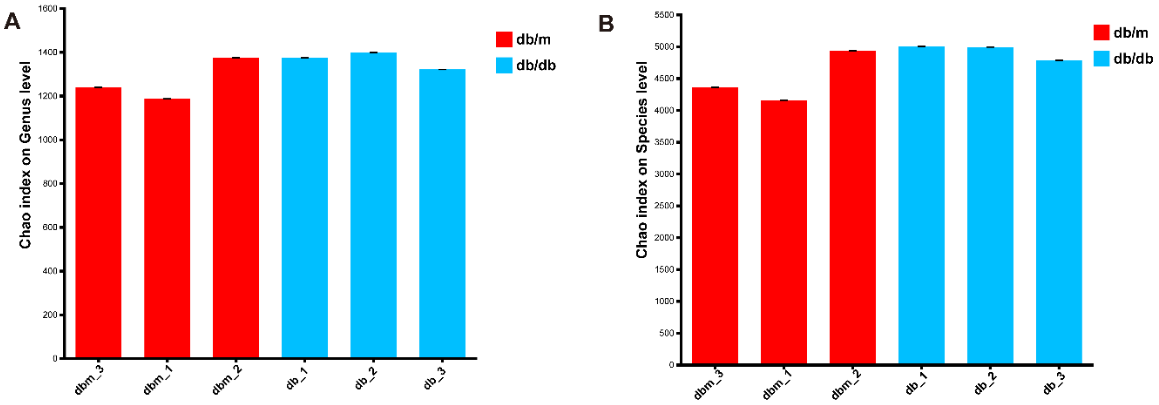

Fig. S3 Column graphs describes the richness of microbial communities/functions in the sample

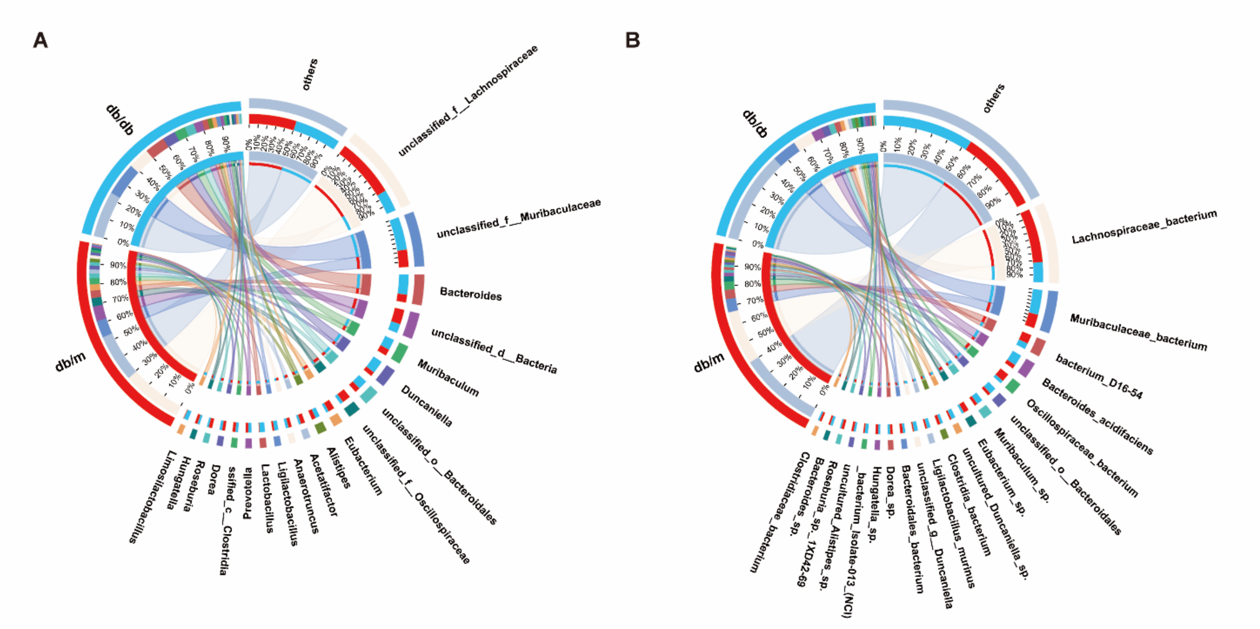

Fig. S4. Circos plot describes the abundance correspondence and proportion between samples and gut microbiota at the genus and species levels

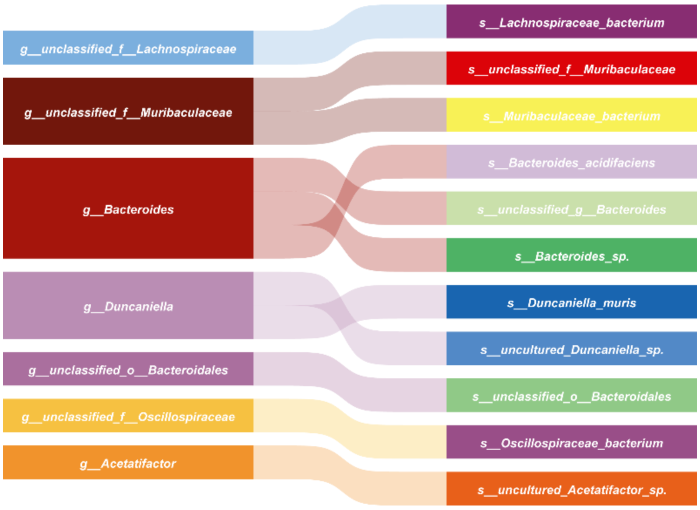

Fig. S5. Sankey diagram to determine the relationship between genus and species

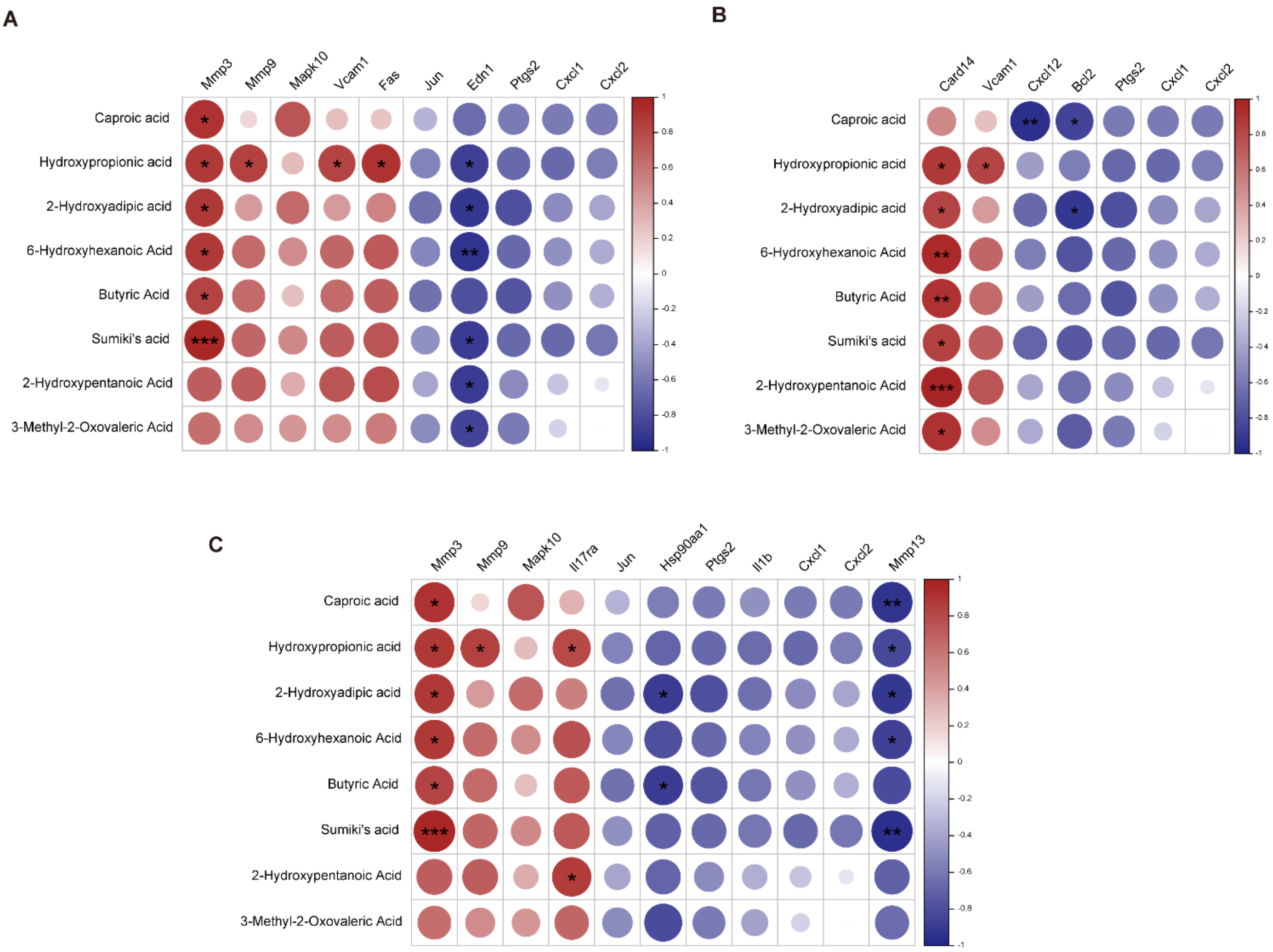

Fig. S6. Correlation heatmap of carboxylic acid and DEGs related to inflammatory pathways (TNF, IL-17, and NF kappa B signaling pathways)
